# The Molecular Basis and Therapeutic Potential of Leukemia Inhibitory Factor in Cancer Cachexia

**DOI:** 10.3390/cancers14122955

**Published:** 2022-06-15

**Authors:** Ruijiang Zeng, Chang Tong, Xiangyang Xiong

**Affiliations:** 1 Department of Biochemistry and Molecular Biology, School of Basic Medical Sciences, Nanchang University, Nanchang 330006, China; 4203118178@email.ncu.edu.cn (R.Z.); changtong@email.ncu.edu.cn (C.T.); 2 Province Key Laboratory of Tumor Pathogens and Molecular Pathology, Nanchang University, Nanchang 330006, China

**Keywords:** cachexia, leukemia inhibitory factor (LIF), cancer, fat loss, anorexia nervosa, muscle atrophy

## Abstract

**Simple Summary:**

The mechanism of cancer cachexia is linked to a variety of factors, and inflammatory factors are thought to play a key role. We summarize the main roles of LIF in the development of cancer cachexia, including promoting fat loss, inducing skeletal muscle atrophy and causing anorexia nervosa. The main aim of this review is to increase the understanding of the effects of LIF in cachexia and to provide new insights into the treatment of cancer cachexia.

**Abstract:**

Cachexia is a chronic metabolic syndrome that is characterized by sustained weight and muscle mass loss and anorexia. Cachexia can be secondary to a variety of diseases and affects the prognosis of patients significantly. The increase in inflammatory cytokines in plasma is deeply related to the occurrence of cachexia. As a member of the IL-6 cytokine family, leukemia inhibitory factor (LIF) exerts multiple biological functions. LIF is over-expressed in the cancer cells and stromal cells of various tumors, promoting the malignant development of tumors via the autocrine and paracrine systems. Intriguingly, increasing studies have confirmed that LIF contributes to the progression of cachexia, especially in patients with metastatic tumors. This review combines all of the evidence to summarize the mechanism of LIF-induced cachexia from the following four aspects: (i) LIF and cancer-associated cachexia, (ii) LIF and alterations of adipose tissue in cachexia, (iii) LIF and anorexia nervosa in cachexia, and (iv) LIF and muscle atrophy in cachexia. Considering the complex mechanisms in cachexia, we also focus on the interactions between LIF and other key cytokines in cachexia and existing therapeutics targeting LIF.

## 1. Introduction

The term “cachexia” originally originated from the Greek words “kakos” and “hexis” and means “in horrible physical condition” [1]. At present, cachexia is considered a multifactorial disease that is associated with malignant and many chronic non-malignant diseases, including kidney disease, heart failure, chronic obstructive pulmonary disease, and cancer [2,3]. Cancer incidence and mortality are among the highest in global epidemiological surveys [2]. In addition, cachexia has been observed in approximately 50% to 80% of advanced cancer patients, especially in patients with metastatic tumors [4,5]. According to an international consensus published in 2010, cancer cachexia is defined as a multifactorial syndrome that is manifested by an ongoing loss of skeletal muscle mass (with or without loss of fat mass), an incomplete reversal of routine nutritional support, and progressive sexual dysfunction. Protein and energy imbalances are common among cachexia patients due to their reduced food intake and abnormal metabolism [6]. This skeletal muscle loss can lead to adverse effects, including increased toxicity from chemotherapy, cancer surgery complications, and increased mortality [7]. Although not all cancer cachexia may be associated with fat consumption, it occurs earlier than skeletal muscle atrophy [8] in some patients and leads to poor quality of life and reduced survival [8,9]. Nausea and premature saturation taste disorder are factors of anorexia that are caused by tumor cachexia [9]. Cachexia can be divided into three stages according to the clinical symptoms of cachectic patients, especially the change in weight: pre-cachexia, cachexia, and refractory cachexia [6]. When refractory cachexia is reached, survival is expected to be no more than 3 months; therefore, early diagnosis and intervention are necessary [6]. Essentially, cachexia-related skeletal muscle atrophy, fat consumption, and anorexia are caused by inflammation and metabolic disorders. The interaction among the tumor, skeletal muscle, and adipose tissue can produce inflammatory factors that promote a cascade response and that disturb the average metabolic balance [10]. Currently, treatment for cancer cachexia mainly includes medication, nutritional intervention, and exercise training, but the efficacy of these treatments is not significant and may even increase the burden on the patient [11]. As a result, it is important to understand the key factors associated with cachexia for new treatment options. Previous studies have identified several cachexia-related cytokines, including interleukin-6 (IL-6), tumor necrosis factor alpha (TNF-α), IL-1β, and the leukemia inhibitory factor (LIF) [12,13,14]. In this study, we focused our attention on LIF.

## 2. LIF and LIF Receptor

**Figure 1:**
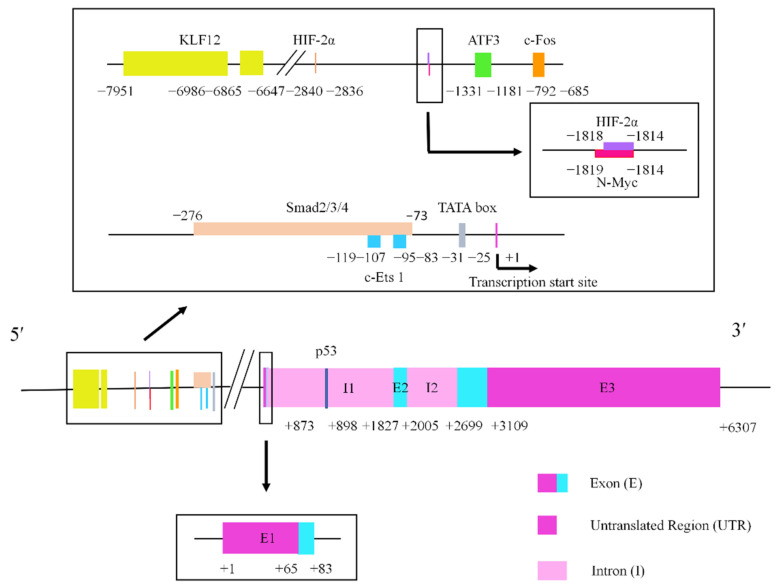
The structure of the cytokine *LIF* gene. The rectangular box exhibits the transcriptional regulatory elements of the promoter. The lengths of the exons and introns are shown in base pairs.

The leukemia inhibitory factor is a secretory glycoprotein with multiple functions [15]. The first study about LIF was published in 1969, when Ichikawa Y identified a protein derived from mice that could inhibit the proliferation of myeloid leukemia M1 cells in vitro on various conditioned media [16]. In 1987, Gearing DP et al. isolated a protein from murine Krebs sarcoma cell cultures that induced the differentiation of mouse myeloid leukemia M1 cells and inhibited their proliferation, thus naming it leukemia inhibitory factor [17]. It has a wide range of biological roles in the neurological, hepatic, endocrine, inflammatory, and immune systems, including the regulation of embryonic stem cell self-renewal, the promotion of embryonic implantation and placental formation, and the stimulation or inhibition of cell proliferation and differentiation, increasing the malignant progression of tumors [15,18,19]. The human *LIF* gene localizes to a 76 kb segment on chromosome 22q12.1-12.2 [20], which consists of three exons, two introns, and a 3.2 kb untranslated region, and yields a 4.1 kb mRNA product [21]. *LIF* genes in humans, mice, and other mammals are highly homologous in their coding and non-coding regions [22], indicating that LIF is a highly conserved molecule. Precursor proteins with 202 amino acids are synthesized by *LIF* mRNA translation, and 22 amino acids are removed from the N-terminal. Finally, the placement of the three disulfide bonds and N-terminal glycosylation steps yield the matured LIF glycoprotein [15,23]. The molecular weight of the non-glycosylated LIF protein is 20-25 kDa, while the glycosylated form ranges from 37 to 63 kDa in weight [24]. In vitro, the biological function of LIF seems to be independent of the degree of glycosylation, but whether glycosylation affects the stability of LIF remains to be determined [23]. Several transcription factors can target *LIF* promoters or enhancers to regulate *LIF* expression. Transforming growth factor beta (TGF-β) activates the *LIF* promoter located at −276/−73 to increase self-renewal in glioma-initiating cells via Smad2/3/4 [25]. Moreover, p53 can regulate maternal reproduction by binding to the intron, located in +873/+898 [26]. The detailed gene structure of *LIF* is shown in Figure 1 [25,27,28,29,30,31,32,33].

LIF belongs to the IL-6 family, which includes oncostatin M, ciliary neurotrophic factor, Charcot–Leyden crystal galactose agglutinin, calcitonin 1, and IL-11 [20,34]. LIF and these cytokines intersect functionally because they share the receptor subunit gp130, which is why they are classified into the same family [22]. In contrast, LIF binds more closely to its specific LIF receptor (LIFR) [7,23]. Existing studies have shown that LIFR can be expressed on many cell surfaces, such as on breast epithelial cells, macrophages, adipocytes, liver cells, and muscle [24,35]. LIF releases via paracrine or autocrine mechanisms, binds to LIFR and the gp130 dimer receptors of the target cells, and then selectively activates signal transduction pathways, including JAK/STAT, MAPK/MEK/ERK, PI3K/AKT, and mTOR, to perform biological functions depending on the cell and tissue conditions [20,36].

## 3. LIF and Cancer-Associated Cachexia

In some cases, LIF has been thought to be a factor in the origin of cachexia. Nude mice carrying melanoma G361 and SEKI cells expressing large amounts of LIF show a cachectic state, whereas nude mice carrying A375 and MEWO cells without LIF expression are not cachectic [37]. In nude mice that have been inoculated with the neuroepithelioma cell line NAGAI, large amounts of LIF are also detected in association with the induction of cachexia [38]. After the surgical treatment of a nude mouse model of gastric cancer cachexia MKN45c185, the initially elevated LIF in plasma is no longer detectable, and cachexia symptoms are eliminated [39]. LIF is also a key factor that is required for the mouse C26 colon cancer cachexia model [40]. Thena, an anaplastic thyroid cancer cell, induces cachexia in mice by expressing higher levels of IL-6, LIF, and TGF-β [41]. In addition, cachexia models that are mediated by highly expressed LIF include the intracerebral injection of human OVCAR3 ovarian carcinoma, A431 epidermoid carcinoma, and GBLF glioma cells in mice [42]. In the organoids produced by pancreatic cancer patients, LIF, IL-8, and growth differentiation factor 15 (GDF15) are prominently up-regulated in cachectic patients [43]. It has been shown that numerous cancers, including pancreatic, colorectal, esophageal, ovarian, renal, gastric, uterine squamous, and testicular cancers, could aberrantly highly express LIF [44]. In addition, because cachexia is a systemic disease, elevated serum LIF is seen as an indicator of poor prognosis for cancer patients, further demonstrating its potential key role in this pathological process [45,46,47]. Studies have shown that some cancer deaths are attributed to cancer-associated cachexia (CAC), such as pancreatic, esophageal, stomach, lung, liver, and colon cancers [2]. Therefore, we suspect that the cachexia in pancreatic, colon, esophageal and gastric cancers can be treated by targeting the abnormally high expression of LIF.

However, the role of LIF in tumors goes beyond cachexia. Tumor-derived LIF can promote the malignant behavior of tumors in an autocrine manner. KRAS mutation could induce LIF expression in human pancreatic ductal adenocarcinoma (PDAC) cells, and LIF inhibits the intracellular Hippo pathway and promotes tumorigenesis by facilitating YAP/TAZ-TEAD interaction and up-regulating the expression of the YAP1 target genes CNTF and ANKRAD. Neutralizing LIF attenuates pancreatic cancer development and improves the sensitivity of cancer cells to drugs [48]. Colon cancer cell-derived LIF can regulate the expression of other cytokines, such as granulocyte-colony stimulating factor (G-CSF) and IL-6, to promote tumor progression [49]. LIF promotes the stem cell properties of osteosarcoma through the NOTCH1 signaling pathway [50] and enhances the growth and invasion of osteosarcoma by activating STAT3 signaling, while blocking STAT3 signaling can inhibit the development of osteosarcoma [51]. LIF derived from breast and colorectal cancer cells can up-regulate miR-21 expression to promote EMT via STAT3 [52]. LIF over-expression promotes colorectal cancer chemoresistance by reducing the level and function of p53 [53]. Increased serum LIF concentrations can increase tumor cell radiation resistance, inhibit DNA repair, and promote tumor recurrence [54]. LIF mediates STAT3 phosphorylation via the autocrine system during TGF-β regulation and promotes PDAC invasiveness through ECM remodeling, maintaining inflammatory fibroblast phenotype activation [55].

Meanwhile, LIF mediates the crosstalk between cancer-associated stromal cells and tumor cells, which is significantly associated with advanced tumor stage, tumor volume, and a short overall survival time [50,51]. LIF is involved in the interaction between cancer cells and cancer-associated fibroblasts (CAF), forming a feedback loop between cancer cells and CAF [56]. Pancreatic stellate cells (PSC) are widely distributed around pancreatic tumors, and their interaction could transform PSC into CAF [55]. CAF-secreted LIF inhibits pancreatic cancer cell differentiation and maintains their stemness, while blocking LIF using neutralizing antibodies and knocking down LIFR both prolong the survival of KP^f^/^f^CL multiple mutant mice [57]. TGF-β can induce CAF to produce LIF, leading to fibroblast activation and the promotion of tumor cell invasiveness [58]. TNF and other inflammatory cytokines that are produced by macrophages in the tumor microenvironment stimulate tumor cells to produce IL-6 and LIF, contributing to tumor growth [59]. LIF could partially control cancer cell immune tolerance by affecting monocytes. Recombinant LIF converts monocytes into tumor-associated macrophage (TAM)-like cells to promote ovarian cancer immunosuppression by inhibiting the expression of the monocyte colony-stimulating factor [60]. LIF blockade results in TAM phenotypic changes, in which C-X-C motif chemokine ligand 9 (CXCL9) expression is elevated, and CD8+ T cells are recruited to the tumor, suggesting that LIF is involved in resistance to immune checkpoint blockade. Neutralizing antibodies to LIF combined with a PD1 immune checkpoint inhibitor could strengthen the immune memory of the host and improve overall survival [61]. Bone marrow mesenchymal stem cells (BM-MSCs) perform a crucial role in supporting the hematopoietic process. It has been shown that BM-MSCs can enhance angiogenesis by releasing LIF to activate the ERK1/2 pathway and induce cancer cells to express vascular endothelial growth factors [62]. In the ovarian cancer microenvironment, cancer-associated mesenchymal stem cells activate the JAK/STAT pathway in ovarian cancer cells by secreting LIF, promoting cancer cell growth, and maintaining their stem cell properties [63]. In addition, our previous research has verified that LIF is highly expressed in cancer-associated adipocytes (CAA) and forms a feedback loop with the CXCLs derived from breast cancer to promote the invasion and metastasis of breast cancer [64].

In summary, LIF promotes tumor progression by being aberrantly expressed in the early stages of cancer and increases the degree of malignancy by affecting the tumor microenvironment. Tumor-derived LIF is a vital initiation factor of cachexia, leading to the progression of cachexia and a poor prognosis for patients with late stages of cancer.

## 4. LIF and Alterations of Adipose Tissue in Cachexia

According to the type of cells present in adipose tissue, it can be classified into white adipose tissue (WAT), brown adipose tissue (BAT), and beige adipose tissue. WAT constitutes the majority of body fat and stores for energy in the form of triglycerides (TG), while BAT generates heat through uncoupled mitochondrial respiration [65]. In recent years, adipose tissue has also been identified as an endocrine organ that secretes a large number of cytokines that are important for the regulation of the body’s systemic metabolism [66]. In cachexia, adipocyte variation has been widely discussed, with studies confirming adipocyte atrophy, suppressed adipocyte differentiation, or the dedifferentiation of mature adipocytes, WAT browning, and extracellular matrix remodeling [67]. Meanwhile, LIF plays a non-negligible role in adipocyte alterations in cachexia, which could bind to LIFR to exert their effects on adipocytes by activating the JAK/STAT and MAPK (ERK1/2) signaling pathways [68].

Morphological changes in adipocyte area size are primarily produced through lipid hydrolysis within the adipocytes [9]. In adipose tissue, TG is mobilized by adipose triglyceride lipase (ATGL), hormone-sensitive lipase, and monoacylglycerol lipase [69]. Among them, ATGL triggers lipolysis, which releases fatty acids from TG [70]. Moreover, lipoprotein lipase (LPL) is the rate-limiting enzyme for triglyceride hydrolysis [71]. Lipolysis, the catabolism of TG, could lead to fat loss, contributing to cachexia [2,72]. Noncoding RNAs such as circPTK2 [73], infection [74], and cytokines such as LIF contribute to lipolysis in cachexia [26]. Available experiments have demonstrated that elevated plasma LIF concentrations are associated with lipolytic enzymes, such as in murine cachexia models bearing SEK1 and NAGAI cells, and that a reduction in LPL activity caused by LIF can regulate lipolysis [75,76]. LIF activates JAK/STAT signaling, mainly the phosphorylation of STAT1 and STAT3, to promote lipolysis via ATGL [77]. Moreover, JAK inhibitors could effectively alleviate adipose loss and improve overall survival, mainly by inhibiting STAT3 phosphorylation [78].

WAT browning is an energy-releasing process that is accompanied by a rise in UCP1 expression that is commonly seen in CAC to meet tumor energy expenditure, which can be mobilized by cytokines, usually IL-6 [9]. WAT browning facilitates CAC due to energy consumption [79]. Moreover, LIF can also promote thermogenesis by facilitating adipocyte browning during exercise [79,80].

**Figure 2:**
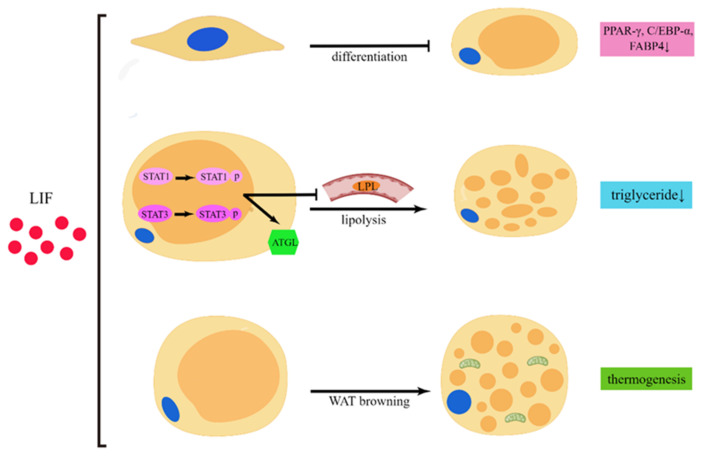
The role of LIF in adipocytes. LIF can activate the phosphorylation of STAT1 and STAT3, increase ATGL to lead to lipolysis, and is associated with decreased LPL activity. Finally, it results in triglyceride loss in adipocytes. LIF also can induce WAT browning, a thermogenic process. Meanwhile, LIF may inhibit preadipocytes’ differentiation to mature adipocytes, as evidenced by the inhibition of PPAR-γ, C/EBP-α, and FABP4 expression (by Figdraw “www.figdraw.com”, accessed on 5 May 2022).

Adipocyte differentiation also has a significant impact on cachexia. The inhibition of adipocyte differentiation has been seen in cachexia mediated by infection [74]; various cytokines [81]; Zinc-α2-glycoprotein [82]; and miRNA, including miR-146b-5p [83], miR-410-3P [84], and miR-155 [85]. LIF may negatively regulate adipocyte differentiation. In preadipocytes, LIFR knockdown results in the reduced expression of the adipocyte differentiation marker genes peroxisome proliferator-activated receptor gamma (PPAR-γ), CCAAT/enhancer-binding protein alpha (C/EBP-α), and fatty acid-binding protein 4 (FABP4) during adipocyte maturation [86]. Moreover, the dedifferentiation of mature adipocytes has also been observed in cachexia and is mediated by tumor-derived proliferin-1 [87] and CircPTK2 [73]. CAA is a type of adipocyte that is formed by the dedifferentiation of the mature adipocytes present in the tumor microenvironment [88]. Some of the alterations that occur in cachectic adipocytes include adipocytes that are small in size or that have decreased TG stores, lower PPAR-γ and C/EBP-α expression, and higher IL-6, TNF-α, IL-1β, and UCP1 expression, which also occur in CAA [89]. We speculate that LIF can promote adipocyte dedifferentiation in the tumor microenvironment and in the case of cachectic adipocytes. The effect of LIF on adipocytes is shown in Figure 2.

## 5. LIF and Anorexia Nervosa in Cachexia

Anorexia nervosa can be seen in various diseases, and its pathophysiological processes mainly manifested as weight loss [90]. Anorexia is closely associated with an imbalance between the orexigenic and anorexigenic neuropeptides in the central nervous system [91] and abnormally elevated levels of leptin [92]. Anorexia nervosa has multiple complications that can affect various organs throughout the body [93]. Many inflammatory cytokines are involved in anorexia nervosa, such as IL-1, IL-6, interferon-gamma (IFN-γ), TNF-α, and leptin [90,94]. Anorexia can be caused by elevated LIF expression, which, according to different studies, decreases food intake and leads to weight loss [92]. Animal experiments have shown that LPS can lead to the up-regulation of LIF expression in the central nervous system, resulting in extensive inflammation and anorexia, and with the combination of LPS and corticosterone, LIF up-regulation is more prominent [95].

Among the causes of cachexia, tumors account for a considerable proportion. Patients with malignant tumors often suffer from cachexia anorexia syndrome during tumor progression [96], tumor metastasis [97], and radiotherapy and chemotherapy [98,99], with the clinical manifestation including losing appetite. Terawaki K et al. revealed that in gastric cancer, the activation of TLR5 signaling by IRAK-1,4 leads to the up-regulation of LIF expression, which in turn leads to anorexia [100]. Arora G et al. showed that in colon adenocarcinoma, LIF-induced anorexia is associated with the activation of JAK/STAT signaling in the hypothalamus [78].

The hypothalamus is essential for regulating feeding behavior and contributes to the development of anorexia, which occurs in various diseases. Current research has confirmed that LIFR is expressed in proopiomelanocortin (POMC) neurons in the arcuate nucleus (ARC) region in the hypothalamus, which can be activated by LIF and then α-MSH released from ARC POMC neurons to induce anorexia [101]. In addition, LIF derived from tumors interacts extensively with the neuropeptides in anorexia [39]. In gastric cancer cachexia, Terawaki K et al. observed that LIF expression was up-regulated, with the elevated expression of orexigenic neuropeptides including neuropeptide Y (NPY), agouti-related protein (AgRP), orexin (ORX), and melanin-concentrating hormone (MCH), and the inhibited expression of anorexigenic neuropeptides, including POMC, amphetamine-regulated transcript (CART), and corticotropin-releasing hormone (CRH) in the hypothalamus [39]. In the SEKI rat cachexia model, the same phenomenon was observed. Although the expression of anorexigenic peptides was down-regulated in both animal models, the bodyweight of the mice was reduced in a cachectic state. We speculate that the effect of neuropeptides may be the self-regulation of the body in response to the anorexia or cachexia effect produced by LIF.

**Figure 3:**
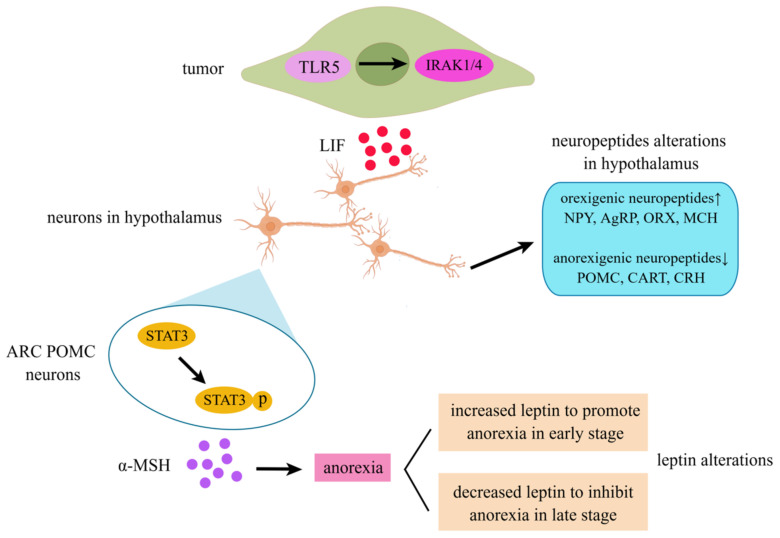
LIF-induced anorexia in cancer cachexia. TLR5 can activate IRAK1/4 to promote LIF secretion in tumors. LIF can facilitate α-MSH secretion from ARC POMC neurons in the hypothalamus that are associated with elevated STAT3 phosphorylation. Additionally, up-regulated LIF is associated with increased orexigenic neuropeptides (NPY, AgRP, ORX, and MCH) and decreased anorexigenic neuropeptides (POMC, CART, and CRH) in the hypothalamus. Leptin increases in the early stage to promote anorexia and decreases in the late stage to inhibit anorexia (by Figdraw, “www.figdraw.com”, accessed on 28 May 2022).

In addition, the progression of anorexia in cachexia caused by LIF is partly influenced by leptin, which is a crucial adipokine, and changes in the adipose tissue or bodyweight can affect the leptin secretion. The leptin released from adipose tissue acts as a signaling mediator from the periphery to the central nervous system, which is essential in maintaining the balance of adipose tissue weight both in physiology and in the pathologic condition [102]. Leptin and LIF have similar structures and can bind to subunit gp130 to activate STAT3 signaling [103]. Notably, a murine animal model confirmed that adipose loss and anorexia induced by LIF are independent of leptin receptor signaling [77]. It was shown that LIF could promote the expression of leptin at an early stage and generate anorexia [104]. However, in the later stages of LIF-induced anorexia, food intake returns to basal levels as serum and adipose leptin expression decreases and hypothalamic STAT3 phosphorylation activation is reduced [77,78,105]. Furthermore, in mice lacking functional leptin (ob/ob) or leptin receptors (db/db), LIF-induced anorexia persisted without recovery [77]. We speculate that anorexia induced by abnormally high LIF expression may be an acute response. At a very early stage, LIF can increase leptin, leading to anorexia and weight loss, but due to adaptive changes in the body, the effects of LIF are disturbed, leptin expression is suppressed, and anorexic behavior is ameliorated. The decreased circulating leptin is an important “natural” systemic response to combat LIF-induced cachexia and therefore may also be important for the mechanisms of potential cachexia therapies. The effect of LIF on anorexia nervosa is shown in Figure 3.

## 6. LIF and Muscle Atrophy in Cachexia

The constant loss and regeneration of skeletal muscle are essential for normal physiological function [106]. In cachexia, the balance between the anabolism and catabolism of proteins is broken, thus promoting lipid oxidation and insulin resistance, inhibiting remodeling, etc. [107]. Patients often suffer from skeletal muscle atrophy [108], especially in CAC. Proinflammatory cytokines such as IL-6, TNF-α, and LIF have been identified to lead to muscle wasting in cachexia [109].

LIF has been observed to be over-expressed in muscle atrophy disease, suggesting that LIF may be related to muscle atrophy [49,110,111]. In colon cancer, LIF can induce muscle atrophy by activating the JAK/STAT [40,112], ERK1/2 [40], and p38 MAPK [112] signaling pathways, resulting in the generation of cachexia. Moreover, the above studies also indicate that inhibiting the JAK/STAT signaling pathways in the myotubes can effectively reduce muscle atrophy, including Ruxolitinib (JAK inhibitor), PIAS3, and the LIF-neutralizing antibody targeting STAT3 and PIAS1 targeting STAT1 [40]. Meanwhile, LIF can also be the target gene of microRNA. MiR-29c can target LIF by combing the area 3′-UTR to down-regulate LIF to inhibit JAK/STAT and MAPK signaling. In the Lewis lung cancer mouse model, the inhibition of lung cancer miR-29c expression resulted in the up-regulation of LIF activity on the myotubes, causing muscle atrophy and cachexia [113].

Under physiological circumstances, both cultured myotubes and muscle can secrete LIF as a response to outside stimulation [114]. However, in pathological conditions, the role of LIF in muscle seems to be paradoxical. LIF can promote the regeneration of muscle atrophy due to denervation [115,116] and injured muscles [117], the proliferation of myoblasts [118], and muscle satellite cells [119]. Clinical trials have demonstrated that exercise inhibits muscle atrophy in cancer patients through the cytokine mediation [120]. LIF expression was significantly up-regulated and accompanied by the down-regulation of myostatin expression, after exercise [121].

**Figure 4:**
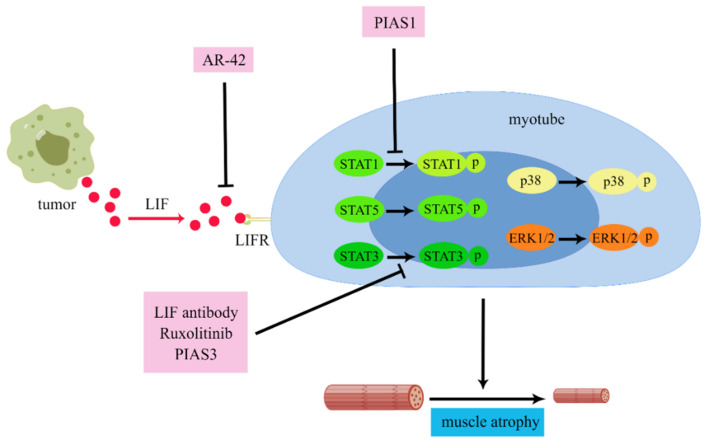
The mechanisms of LIF in cachexia-induced skeletal muscle atrophy. LIF targeted at myotubes can activate STAT1, STAT3, STAT5, ERK1/2, and p38 signaling. PIAS1 can block STAT1 phosphorylation to inhibit muscle atrophy. The LIF antibody, Ruxolitinib (JAK inhibitor), and PIAS3 can block the phosphorylation of STAT3 to inhibit muscle atrophy. Additionally, AR-42, a histone deacetylation inhibitor, can block LIF to treat CAC-induced muscle atrophy (by Figdraw “www.figdraw.com”, accessed on 5 May 2022).

Overall, LIF can exert multiple effects on skeletal muscle cells under different conditions [122]. This seems to be related to the crosstalk of other factors in the cachexia, which needs to be further elucidated for better treatment to improve the prognosis of tumor patients. The effect of LIF on skeletal muscle atrophy is shown in Figure 4.

## 7. LIF and Other Crucial Factors of Cachexia

Although LIF plays a significant role in cachexia, the pathological process of cachexia is not achieved by LIF alone. Studies at this stage suggest that cancer cachexia is a cross-linked network of inflammatory factors [123]. When a particular inflammatory factor is over-activated, it activates other cachectic factors through this network, causing a cascade reaction that acts on different target organs to form the final pathological state of cachexia. Currently recognized cachexia factors include IL-6, IL-1α, IL-1β, TNF-α, IFN-γ, and GDF15, which are related to the presence of LIF [108]. In rLIF-driven cachexia mouse models, the mRNA levels of IL-6 increased 50-fold, and in both models, this was associated with anorexia and fat loss [108]. In vitro, the lipolysis caused by TNF-α and IL-6 was two-fold and three-fold higher than that caused by LIF, respectively [59]. The separate stimulation of TNF and IL-1β promoted the secretion of LIF and IL-6 in a cachexia study with tumor-bearing mice [124], resulting in a synergistic effect [125]. In addition, LIF can promote the secretion of IL-1α, but the mRNA content does not increase [126]. It is worth noting that there seems to be a consensus that the activation of IL-6 expression follows the up-regulated expression of LIF, thereby promoting the occurrence of anorexia and cachexia syndrome in tumor patients [78,120,121]. However, some research has not detected the elevated expression of IL-6 accompanied by the over-expression of LIF secreted by the tumor in animal models [110,122,127].

Studies have confirmed that TNF-α and IFN-γ are essential cachexia mediators. They can cause a reduction in food intake and bodyweight in mice in sub-lethal amounts [128]. In chordoma, TNF-α has a secretory cycle-promoting effect with LIF that is associated with a synergistic effect and reduced overall patient survival [129]. However, the relationship between LIF and IFN-γ remains unclear in cachexia. IFN-γ can modulate the impact of TNF-α by reducing LIF expression [130], and yet, in encephalitogenic cells, inducing IFN-γ can increase the level of LIF [131].

The most vital role of GDF15 is to regulate energy homeostasis, which can affect normal appetite and cause cachexia when its expression is elevated due to tumors [132]. GDF15 can successively activate the ERK pathway, increase LIF expression through c-Fos binding to the *LIF* promoter region (−685/−792), and then promote the phosphorylation of STAT3, thus expanding the stemness of glioma cells [33].

Overall, LIF can lead to cachexia through lipolysis, anorexia, and skeletal muscle atrophy in tumors. We summarize the relevant molecular basis of cachexia-associated transcriptional regulation of the LIF gene in Table 1 and genes or enzyme activity regulated by LIF in Table 2.

## 8. LIF and Therapeutics in Cachexia

Numerous studies have demonstrated that tumors with abnormally high LIF expression not only cause more rapid tumor proliferation and greater malignancy in migration and invasion in the early stages but that they also induce cachexia in the late stages, resulting in fat loss, anorexia nervosa, and skeletal muscle atrophy. Given this, targeted LIF may reduce tumor progression and the development of late-stage malignancy, allowing patients to have a better quality of life.

In animal experiments, inhibiting JAK, downstream of LIF, can effectively alleviate cachexia [40,78]. At present, histone deacetylation inhibitor AR-42 has been observed to impede muscle atrophy by blocking multiple pro-cachexia drivers, including LIF, to ameliorate cachexia [112]. However, in some cases, LIF can also facilitate the proliferation of muscle cells. Completely suppressing the effect of LIF is not the best option. Therefore, a more thorough study of the dual role of LIF in muscle proliferation and atrophy is warranted. In addition to drug therapy, some attempts have been made to remove LIF from plasma by physical adsorption to treat cachexia, but these studies failed [133].

In addition, blocking LIF to improve anorexia nervosa and fat loss should be an effective treatment. The application of Ghrelin resulted in an increase in bodyweight in LIF-induced mice cachexia and a corresponding increase in serum leptin levels [134]. In addition, Guo et al. demonstrated that LIFR knockdown can decrease LIF-induced lipolysis and contribute to the reduction of browning markers, ultimately leading to an increased fat mass and bodyweight in mice [135]. Notably, lipolysis and anorexia are indirectly related. Decreased protein intake and insufficient energy intake due to anorexia can compensate for energy metabolism through lipolysis. However, Arora GK et al. observed that there was a significant reduction in both fat and bodyweight in mice injected with rLIF in the absence of differences in food intake compared to the control group, suggesting that LIF-induced lipolysis may have mechanisms that are independent of changes in feeding [77]. This means that simply improving the symptoms of anorexia does not stop cachexia from continuing, resulting in the continuation of fat loss. Weight maintenance is the ultimate goal in the treatment of cachexia; therefore, we need to consider the therapeutic potential of LIF in cachexia in a holistic manner. Improving fat loss, anorexia, and skeletal muscle atrophy during the course of cachexia, as well as treating the tumor, the cause of the cachexia, could better improve patient prognosis.

## 9. Conclusions

Overall, the available studies have confirmed that LIF can lead to weight loss through skeletal muscle atrophy, fat loss, and anorexia nervosa, which contributes to the progression of cachexia. However, there are still some interesting and important questions that deserve further exploration.

First, in recent years, immunotherapy has been applied to cachexia. Immune cells in the tumor microenvironment, including TAM, tumor-infiltrating lymphocytes, and tumor-associated neutrophils, are considered to be capable of secreting large amounts of circulating factors such as IL-6 and IL-1 to promote the malignant progression of cachexia [136]. It has been shown that abnormally high LIF expression in the tumor microenvironment can promote macrophage aggregation, which could secrete IL6 and IL-1α to induce muscle atrophy and cachexia [61,137]. It is well known that most patients with advanced PDAC are in a cachectic state [138], perhaps because LIF is highly expressed in the tumor cells, macrophages, and mast cells in the tumor microenvironment of PDAC patients [139]. In animal experiments, the removal of macrophages prevented and alleviated cachexia [140]. Therefore, perhaps we can achieve a similar effect by targeting LIF inhibition. Unfortunately, there is little research on LIF in this immunotherapy for cachexia, and more in-depth studies can provide more methods for treating cachexia.

Second, the generation and development of cachexia is a highly complex network of inflammatory cross-linkages. Identifying the most crucial malignancy triggers according to disease type and blocking the cascade of inflammatory pathways at the source may be valuable for treating this pathological process. As previously stated, LIF is one of the sources in some diseases. In current clinical studies, good progress has been made in improving cachexia with therapies that counteract key cachexia factors such as IL-6, IL-1β, and IL-1α [14,141,142]. Recently, EC330, a targeted inhibitor of LIF, has been observed to significantly inhibit the malignant behavior of tumors at both the cellular and animal levels and may also be used in cachexia-related studies [143].

Finally, LIF research in this area is still focused on the cellular and animal levels. More needs to be invested in research, especially clinically relevant trials. LIF plays a broad role in cachexia, so we believe that targeted LIF has excellent potential for treating cachexia. Clinical trials may bring hope to patients. Of course, cachexia treatment is multidimensional, and it is not the best option to look at only one aspect of the problem. However, a more thorough study of LIF and therapeutic drugs in cachexia would definitely complement current treatments.

## Figures and Tables

**Table 1:** Transcriptional regulation of the *LIF* gene.

Cytokine, Transcription Factor, or microRNA	LIF Expression	Cell Type and Context	References
HIF-2α	up	RKO and HCT116 colorectal cancer cells	[31]
TNF, IL-1β	up	4JK macrophage derived from tumors	[59]
TGF-β	down	mouse PSCs, KPC primary tumor cells	[55]
IL-1	up	mouse PSCs, KPC primary tumor cells	[55]
TGF-β	up	fibroblasts, SCC12 carcinoma cells	[58]
GDF15	up	glioma stem cells	[33]
KRAS	up	BxPC3 and SW1990 pancreatic cancer cells	[48]
EBV-encoded protein latent membrane protein 1	up	nasopharyngeal carcinoma (NPC) serum samples	[54]
TLR5, IRAK-1/4	up	85As2 gastric cancer cells	[100]
CXCLs	up	CAA	[64]
miR-29c	down	Lewis lung carcinoma cells	[113]

**Table 2:** Genes, transcription factors, or enzymes regulated by LIF.

Genes, Transcription Factors, or Enzymes	Regulation by LIF	Function	References
YAP/TAZ-TEAD	up	suppress the Hippo pathway in PDAC cells	[48]
IL-6, G-CSF	up	mediate cachexia in colon carcinoma cells	[49]
NOTCH1, HEY1, HEYL, HES1, KRT19	up	keep stem cell-like properties of osteosarcoma	[50]
miR-21	up	promote EMT of breast and colorectal cancer cells	[52]
p53	down	promote chemoresistance of colorectal cancer	[53]
genes of p70S6K signaling, mTOR	up	promote NPC cell growth	[54]
CXCL9	down	inhibit CD8+ T cell tumor infiltration	[61]
STAT3	up	promote ovarian cancer cell growth	[63]
CXCLs	up	promote breast cancer cell invasion and metastasis	[64]
IL-6	up	induce cachexia	[2]
STAT1/3, ATGL activity	up	induce lipolysis	[77]
LPL activity	down	induce lipolysis	[75,76]
PPAR-γ, C/EBP-α, FABP4	down	inhibit adipocyte differentiation	[86]
α-MSH	up	induce anorexia	[101]
leptin	up	induce anorexia	[77,104]
NPY, AgRP POMC, CART	up down	compensate for anorexia	[39,101]
p38	up	induce muscle atrophy	[112]
STAT1/3/5, ERK1/2	up	induce muscle atrophy	[40]
mTORC1	up	induce myotube protein synthesis	[12]
